# Allergen-Specific Immunotherapy in Food Anaphylaxis

**DOI:** 10.1097/WOX.0b013e318165b9c1

**Published:** 2008-03-15

**Authors:** Regina Kerzl, Martin Mempel, Johannes Ring

**Affiliations:** 1 Department of Dermatology and Allergy, Biederstein, Technische Universität München (TUM), Munich, Germany; 2 Division of Environmental Dermatology and Allergy GSF/TUM, GSF National Research Center for Environment and Health, Neuherberg, Germany

**Keywords:** allergen-specific immunotherapy, food allergy, long-term effect

## Abstract

Specific immunotherapy (SIT) protocols for nutritional allergens have only recently been established with a focus on oral allergy syndrome because of pollen cross-reacting antibodies. For these patients, a substantial number of studies have been published suggesting benefits from SIT. The situation in true anaphylaxis to food allergens such as peanut allergy is more complex, and therapeutic strategies are based on individual protocols rather than controlled studies. However, in defined cases, SIT represents a promising approach for a durable protection from life-threatening risks after accidental ingestion.

## 

Anaphylaxis to food is recognized as a common worldwide problem, and its incidence seems to increase. In the past years, investigations of allergic food proteins and immunologic responses have provided new knowledge on strategies to prevent food-induced anaphylaxis, one of them being the allergen-specific immunotherapy (ASIT) [1]. In contrast to the prevalent "gold standard," the avoidance of the responsible food, or the application of pharmacological regimens, which target only the symptoms without affecting the allergic pathogenesis, it represents the only curative and specific approach for type I allergy [2,3].

## Mechanisms of ASIT

The mechanisms by which the desensitizing effect is achieved includes mainly the modulation of T cells and B cells, especially by the generation of allergen-specific T-regulatory cells leading to suppressed T-cell proliferation and an enhanced T_h_1/T_h_0 cytokine response against the allergen. Specific immunotherapy leads to an effective reduction of the threshold for mast cell and basophil activation and to a significant in-crease in allergen-specific immunoglobulin G4 (IgG4), IgG1, and IgA, and a decrease in IgE in the late stage of the disease [4]. As a result, successful ASIT does not only rise the allergen concentration needed to induce immediate- or late-phase reaction in the target tissue, but also diminishes the response to nonspecific mucosal stimulation [5].

## Problems of ASIT in food anaphylaxis

Although ASIT is a widely used immunomodulatory treatment of different allergic diseases, for example, seasonal allergic rhinitis, asthma, or insect venom hypersensitivity, there are several problems concerning ASIT in food anaphylaxis. In contrast to the diseases mentioned previously, the clinical relevance is not given without restrictions. The expression of food allergy varies with age, as for example, the sensitization against cow's milk's (CM) protein or egg occurs during the first 2 to 3 years of life, but is most likely to vanish within adolescence [6]. Because of this, the indication for SIT is rather doubtful because avoidance of the responsible food is often manageable.

However, there are situations where avoidance is not that easily possible. Undeclared allergens in processed products represent a major health problem for sensitized persons. Peanut flour and gluten are known as potent food allergens. When evaluated with enzyme-linked immunosorbent assay, the hidden presence of gluten and peanut in Polish products was detected in 4 (11.8%) of 34 processed foods (corn crisps, wafers, cereal bar, and halva) and 5 (13.5%) of 37 foodstuffs (cornflakes, corn crisps, bouillon cube, vegetable soup for infants, curry powder) contained undeclared gluten [7,8]. Until recently, most food-control authorities were not in the position to monitor hidden allergens and to take legal measures against their presence in foodstuffs [9]. Under this aspect, immunotherapy would be a great benefit for people with severe anaphylaxis after ingestion of the acclaimed products.

In addition to the theoretical difficulties in the indication for ASIT therapy, there are major obstacles when it comes to the correct realization of this treatment. This is mainly the lack of standardized extracts from either native proteins or recombinant allergens. Moreover, there are several essential requirements that have to be guaranteed for the development of safe and efficient ASIT vaccines such as the reproducible clinical benefit, the lack of side effects, easy application, and a good onset-to-clinical success ratio [2,4,10].

## Prevalent use of ASIT on food allergy

### Standardized schemes of ASIT in pollen allergy and pollen-related oral allergy syndrome

#### Pollen allergy

The efficacy of ASIT in pollen allergy is already well established [11]. Besides the conventional standards for ASIT with grass and tree pollen extracts like birch,[12] alder, and hazel, a new focus has been reported toward the establishment of ASIT to olive pollen. In the Mediterranean countries and some areas of North America, allergy to *Olea europaea *pollen is a major health problem [13]. The prevalence of pollinosis depends on the cultivation intensity and on the percentage of sensitization to olive pollen in individuals with allergic rhinitis varying from 15.2% in the Montpellier area [14] to greater than 80% found in the southern Spanish province Jaén [15,16]. In addition, severe anaphylactic reactions to pollen products such as honey and jelly have been described recently [17-19]. Besides, as the IgE-binding pattern of olive pollen is complex and diverse, some olive allergens like Ole e 9 and Ole e10 also exhibit IgE cross-reactivity with proteins from latex and vegetable foods, such as tomato, kiwi, potato, and peach [20,21]. Patients with allergies to these materials would benefit from ASIT against olive pollen, which is already the current standard of treatment to induce hyporesponsiveness by applying injections of increasing amounts of allergen extracts [22,23]. Although the use of ASIT with standardized olive pollen extracts is considered to be an effective method to reduce symptoms of a type-I allergic reaction, there is still the risk of adverse reactions because of the high diversity of the allergenic components of olive pollen. Among them, 10 different allergens have been characterized by now,[24] and it seems to be an important deficiency in the current standardization methods, that the amount and affinity of individual IgE antibodies directed against a particular allergen, either major or minor, are not adequately taken into account. A solution to increase the safety of allergen vaccines could be the reduction of minor allergen content,[15] and for prevention of allergic reactions, a prophylactic intranasal treatment with recombinant allergen fragments might be a promising attempt [25]. However, further studies will be required to assess new strategies and to fully determine the underlying mechanisms.

#### Pollen-related oral allergy syndrome

Specific immunotherapy is not only an established method used in pollen-induced allergies, but also in pollen-related allergic reactions to nonpollen products. Up to 70% of individuals with pollen allergy perceive symptoms like immediate itching in mouth and throat or mild-to-moderate angioedema after ingestion of fresh fruits of the Rosaceae family, nuts, or vegetables, also referred to as the oral allergy syndrome (OAS) [26,27]. This phenomenon is caused by cross-reactive IgE antibodies that recognize homologous allergens in birch pollen and fruits,[28-30] the primary sensitizer being the major birch pollen allergen Bet v 1. Its fruit homologues are more liable and sensitive to processing in the stomach and therefore lack sensitization directly. Selective recognition of epitopes on fruit or vegetable homologues of Bet v 1 are found to be extremely rare,[31] so that the spectrum of IgE specificities is broadest against the pollen allergen. Taking these aspects into account, several studies have investigated the effect of ASITwith tree pollen on OAS. It has been a long-standing debate whether immunotherapy for inhalant allergies indeed has a beneficial effect on associated food allergies, but despite some studies with limited effect/negative results,[32,33] a positive impact has been demonstrated in most cases, with improvement of the OAS from 50% up to a complete loss of symptoms in subjects with monosensitized birch pollen allergy confronted by an open apple challenge [34]. Reasons for the different outcomes might be the high dose dependence of the effect of ASIT and a strong difference in the concentration of the major allergen Bet v 1 in the commercial extracts or the existence of other less well-defined cross-reactive allergens, which are not present or sufficiently enough in the birch pollen extracts [35]. According to these findings, further studies will be required to determine whether the most effective treatment of OAS can be achieved using ASIT with corecognized inhalant allergens or through cotreatment with purified pollen-related food allergens to guarantee a long-lasting effect [36].

### Individual schemes of SIT

#### CM allergy

In contrast to the standardized schemes of SIT for pollen-induced and pollen-related allergies mentioned previously, in certain food allergies, individually adapted schemes have to be applied. Especially in protein allergy, like CM allergy in young children with atopic eczema, the variable response of each patient to the different allergen components may influence the potential clinical outcome. Therefore, the mode of application of ASIT is a very delicate matter and not seldom has to be interrupted and discontinued because of the occurrence of uncontrolled side effects that might indicate possible differences in the mechanism, which are known for classical immunotherapy with airway allergies. With respect to the fact that prolonged elimination diets are not without risks, such as deficiency and growth retardation,[37] eating disorders, impaired psychosocial development,[38,39] or even severe anaphylactic reactions,[40] SIT has become a successfully used alternative, the major aim of which is not to alter the cause of food allergy, but to induce a tolerance toward the incriminated food and to increase the threshold dose to elicit allergic symptoms in case of continuous exposure. One current scheme for CM desensitization is to administer increasing amounts of CM starting from 1 drop of whole CM diluted 1:25 with water corresponding to approximately 0.06 mg of CM protein. Afterward, the doses are doubled first every 7 days until day 70, then subsequently every 16 days, until a daily intake of 200 mL is achieved (normally in about 6 months) [41]. Besides, there are many different proposals how to receive successful results,[42,43] their optimal adaptation for each individual patient probably being the best way to guarantee an effective clinical outcome. However, these schemes of daily application differ from the classical way of ASIT. To allow exposure to a gradually escalating dose of a specific allergen to decrease allergic and inflammatory responses, a buildup phase with once weekly applications is followed by a maintenance phase with monthly repetitions that has to be continued for several, normally 3 to 5, years [44]. Daily application requires far more compliance of the patient and thus might bear a higher risk of adverse reactions if interrupted for some days. To most, applying these schemes is a very time-consuming procedure and demands discipline and experience. Despite previous tolerance, on reexposure to the allergen, moderate systemic reactions might easily occur if a certain maintenance dose is not guaranteed [45]. Oral tolerance induction by ASIT in CM allergy is therefore not to be regarded without limitations and has to be discussed individually for each patient.

#### Nonstandardized/experimental approaches

The current information on custom-made protocols for ASIT in severe food anaphylaxis is rather scarce. In cases in which avoidance is not possible, ASIT should, however, be considered. Most of the data come from approaches in severe peanut allergy, which is associated to life-threatening symptoms to a high degree. Although ASIT in peanut allergy has been considered extremely difficult, there are several reports encouraging this strategy [46-48]. We have undertaken an individualized ASIT approach to an adult patient with severe kiwi anaphylaxis who was effectively desensitized by a sublingual extract from fresh kiwi pulp. Although the whole procedure is extremely time consuming as one might have to start with a concentration of one tenth of the lowest positive concentration in skin prick test,[49] recent findings could demonstrate the efficacy and long-lasting protective effect of the applied ASIT [50]. This effect was also paralleled by a sub-stantial induction of kiwi-specific IgG4 [11].

In the future, the use of specific IgE antibodies such as omalizumab could be an interesting point of research to reduce the occurrence of adverse systemic reactions in immunotherapy. Some recently published studies outline the positive effects of the monoclonal anti-IgE antibody as a pretreatment in peanut allergy,[51] rush immunotherapy for ragweed-induced seasonal allergic rhinitis,[52] or in injection immunotherapy with a moderate dose of house dust mite extract in house dust-sensitive adults with atopic eczema [53].

In addition, new genetically engineered major allergens from food sources will enable better standardized and hopefully better tolerable protocols.

## Conclusion

Taken together, immunotherapy in food anaphylaxis is a possible option but not always easily applicable because of the risk of side effects. Several standard schemes exist for pollen-induced and pollen-related food allergy, but still for some products, such as CM or peanut, individual procedures have to be applied to guarantee a successful tolerance induction. Even if only a few studies allow assumption of a generally long-lasting protective effect of ASIT by now, the results provided up to now are quite encouraging [54]. To guarantee a safer and more convenient use of ASIT in the future, more studies will be necessary.
